# Blood bank trends in Kuwait: five-year analysis of donations and investigations

**DOI:** 10.3389/fpubh.2025.1714693

**Published:** 2025-12-15

**Authors:** Dalal Almuaili, Rabaa Abdullah, Anwar M. Al-Awadhi

**Affiliations:** 1 Department of Medical Laboratory Sciences, College of Allied Health Sciences, Kuwait University, Jabriya, Kuwait; 2 Ministry of Health, Sulaibikhat, Kuwait

**Keywords:** Kuwait Central Blood Bank, Blood utilization, Antenatal testing, Alloantibodies, Transfusion transmissible infection (TTI)

## Abstract

**Background:**

Reliable national transfusion services require continuous surveillance of donation activity, inventory losses, transfusion-transmissible infection (TTI) screening, and immunohematology workload. Kuwait’s centralized service is coordinated by the Kuwait Central Blood Bank (KCBB).

**Study design and methods:**

We performed a retrospective analysis of officially requested KCBB annual reports (January 2019–December 2023). Collated variables included donation volumes, donor sex and ABO/RhD distribution, discarded components, and NAT screening outcomes for HBV, HCV, and HIV. Patient-side indicators comprised total immunohematology samples, antibody screening/identification, antenatal testing, and alloantibody profiles. The analysis was descriptive, presenting distributions and temporal trends without inferential testing.

**Results:**

Donations averaged ~82,000 annually, with a decline during the COVID-19 pandemic (2020–2021) and recovery to ~85,000 in 2023. Male donors accounted for >85% of donations. O RhD^+^ positive (38.7%) and B RhD^+^ positive (23.7%) were the most common blood groups, while RhD-negative donors comprised 8.6%. Wastage varied yearly, predominantly impacting fresh frozen plasma. NAT-reactive TTI prevalence remained low: HBV 0.06–0.11% (60–110 per 100,000), HCV 0.03–0.08% (30–80 per 100,000), and HIV 0.02–0.05% (20–50 per 100,000) annually. Immunohematology workload (total samples and test activity) fell during 2020–2021 and increased again by 2023. A wide spectrum of clinically significant alloantibodies was identified, most frequently within the Rh, Kell, and Kidd systems, with additional MNS and P1PK specificities. Among antenatal samples, antibody positivity averaged 3.8% (peak 4.7% in 2021); anti-D predominated, followed by anti-K.

**Discussion:**

KCBB data highlight persistent male predominance among donors, RhD-negative scarcity (8.6%), and variable component wastage, especially FFP. These findings support targeted donor-recruitment strategies, obstetric–transfusion planning for RhD-negative supply, and strengthened inventory management to improve resilience and safety of Kuwait’s blood supply.

## 1 Introduction

Blood transfusion services are a cornerstone of modern healthcare, underpinning surgery, trauma care, hematology–oncology, and chronic disease management. Sustaining an adequate national supply requires continuous surveillance of donation patterns, donor demographics, ABO/RhD distribution, and viruses responsible for transfusion-transmissible infections (TTIs) to align collections with clinical need and maintain safety (1).

Globally, donation trends reflect sociodemographic factors and health-system capacity, and they were notably disrupted by COVID-19 pandemic, which affected mobile drives, donor attendance, and operations across many services (even where annualized volumes ultimately rebounded). These shocks exposed vulnerabilities in collection logistics, donor mobilization, and inventory buffers (2).

Planning also depends on population blood group distribution because ABO and RhD prevalence shape inventory mix and the ability to meet demand for specific phenotypes. National and regional programs therefore track group frequencies and align collection targets and component processing accordingly (1).

Component discard (wastage) remains a persistent operational challenge. Studies report wide variation by product and setting, with fresh frozen plasma (FFP) and platelets frequently over-represented among discards; leading causes include inappropriate/duplicate orders, failure to reissue before expiry, and temperature/thaw-related issues. These patterns highlight opportunities for process redesign and data-driven inventory control (3, 4).

Concurrently, donor screening has driven TTI risks to historically low levels in high-income settings (e.g., Canada: residual risk ≈ 1 in 19.7 million for HIV, 1 in 41.5 million for HCV, and 1 in 2.9 million for HBV), underscoring that today’s primary constraints are often availability and logistics rather than biological safety (5).

Beyond focusing on donors, effective health-system planning also requires visibility into the transfusion recipients. This includes understanding the immunohematology workload generated by crossmatching, antibody screening, and compatibility testing. Monitoring alloantibody prevalence in referred samples and antenatal antibody screen positivity informs crossmatch complexity, phenotype/antigen-negative inventory, and policies to prevent Hemolytic Disease of the Fetus and Newborn (HDFN). Recent guidelines reinforce systematic antenatal grouping, antibody testing, and early referral pathways for clinically significant antibodies (6, 7).

Kuwait, with a population of ~4.8 million, relies on Kuwait Central Blood Bank (KCBB) as its national transfusion center. Operating under the Ministry of Health, this centralized model facilitates system-wide quality management, inventory coordination, and policy implementation. KCBB also serves as the sole national facility for advanced immunohematology services, including but not limited to antibody identification, extended red cell phenotyping, and antenatal testing.

Ensuring resilience and efficiency in blood services requires input from experts beyond blood collection, processing, and laboratory teams. Modern transfusion systems increasingly draw on fields such as operations research, supply-chain management, and data analytics/AI to improve donor recruitment, optimize inventory levels, coordinate redistribution between facilities, and anticipate shortages. These approaches are now well documented in the health-services and operations literature (8–10).

Against this backdrop, we analyzed 5 years (2019–2023) of KCBB data on (i) donations (trends, ABO/RhD, demographics), (ii) component discard, (iii) donor screening outcomes for HBV/HCV/HIV, and (iv) recipient immunohematology workload including total samples received, test types (e.g., antibody screen/ID), antenatal samples, and alloantibody prevalence to provide a comprehensive, system-level picture to inform policy and logistics in Kuwait.

## 2 Methods

### 2.1 Data source

The datasets analyzed for this study were derived from the official annual reports of the Kuwait Central Blood Bank (KCBB) covering January 2019 to December 2023. These reports are not publicly available online but can be requested in printed form from the KCBB through formal official channels or from the corresponding author.^1^ They provide national summaries of donation activity, donor demographics, blood group distribution, component utilization and discard, transfusion-transmissible infection (TTI) screening, as well as patient-related immunohematology workload, including antibody screening/identification requests, antenatal testing, and alloantibody prevalence.

### 2.2 Data management and variables

All available data from the annual reports were collated into a unified dataset using Microsoft Excel (Microsoft Corp., Redmond, WA, United States). Donor-related variables included the annual number of donations, sex distribution, and ABO/RhD frequencies. Screening outcomes captured the prevalence of HBV, HCV, and HIV reactivity. Component utilization was assessed by quantifying discarded blood components, specifically leukoreduced red blood cells (LR-RBCs), FFP (It should be noted that we primarily reported data for FFP prepared with CPD anticoagulant), and platelets. Patient workload indicators included the total number of samples processed for immunohematology, requests for antibody screening and identification, the number of antenatal samples tested, and the prevalence of clinically significant red cell antibodies.

### 2.3 Statistical analysis and calculations

Descriptive statistics were conducted using GraphPad Prism version 10 (GraphPad Software, San Diego, CA, United States). ABO and RhD frequencies are presented as percentages with mean ± standard deviation (SD). TTI prevalence is expressed as the proportion of reactive donations per year relative to total donations. Antibody prevalence is reported as a percentage of positive results relative to total samples tested.

Prevalence of transfusion-transmissible infections was calculated as the number of NAT-reactive donations per 100,000 donations, consistent with international reporting practices. Both percentage prevalence and rates per 100,000 donations are presented (11).

Discard rates were calculated by dividing the number of discarded units reported in the annual summaries by the total number of prepared or collected units, multiplied by 100.

### 2.4 Editorial and research assistance

Artificial intelligence assistance (ChatGPT, OpenAI) was used to support text editing, language refinement, and retrieval of background references. All data extraction, statistical analyses, and interpretation were performed by the authors. AI input was limited to improving clarity of presentation and drafting narrative sections.

### 2.5 Ethics approval

Ethical approval was granted by the Health Sciences Centre Ethics Committee (HSCEC) at Kuwait University (VDR/EC-2025-118). As the analysis relied on aggregated, publicly available data, individual donor consent was not required.

## 3 Results

### 3.1 Donor ABO and RhD distribution

**Figure 1:**
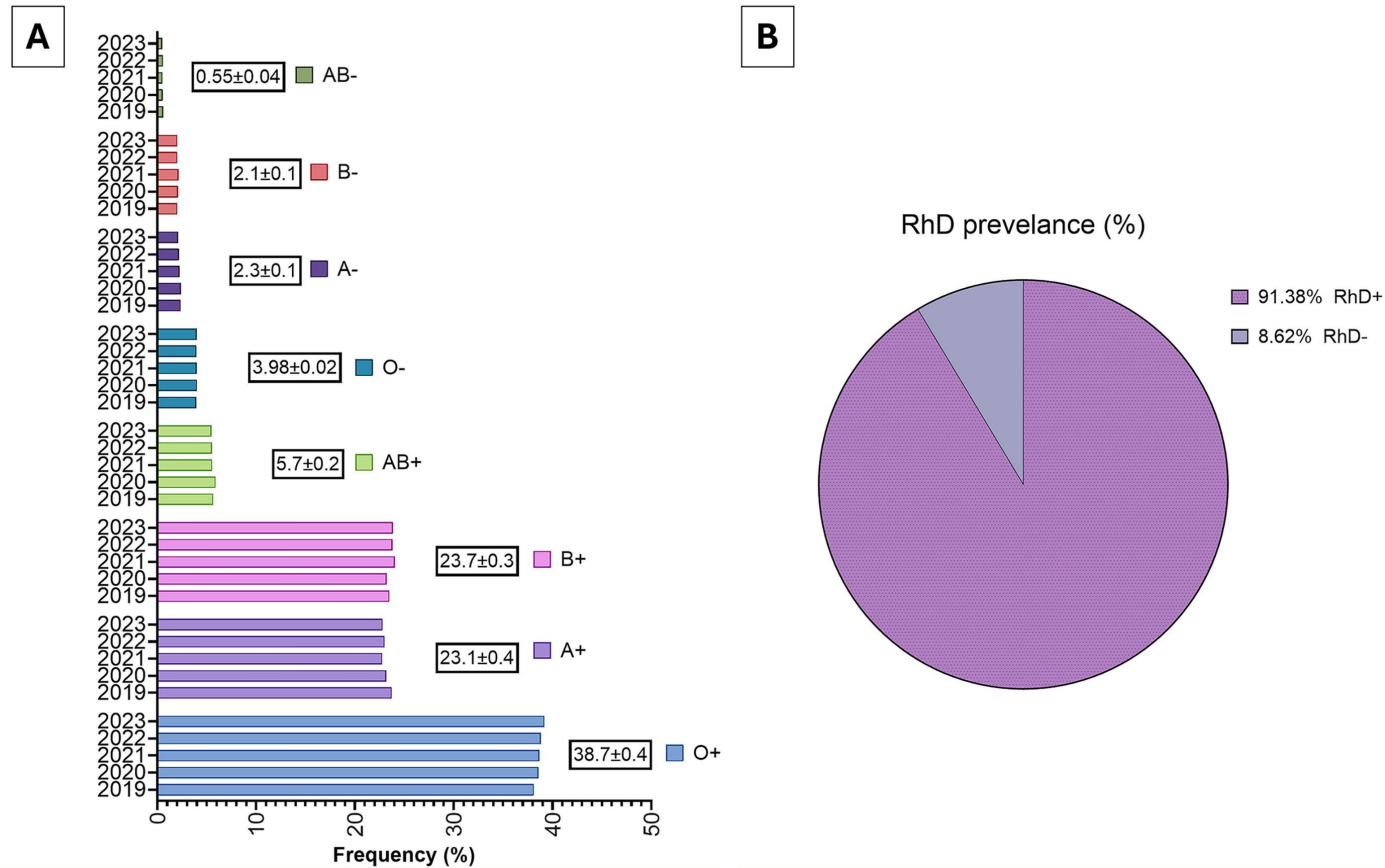
Distribution of ABO and RhD blood groups among blood donors in Kuwait, 2019–2023. **(A)** Annual prevalence of ABO and RhD blood groups among all donations collected at Kuwait Central Blood Bank between 2019 and 2023 is shown, including mean ± SD. O RhDpositive (38.7%) as the most common group, and AB-negative as the least common. **(B)** The overall prevalence of RhD phenotypes is summarized in the pie chart, showing 91.4% RhD-positive and 8.6% RhD-negative donors.

The ABO and RhD distribution among blood donors remained stable across the study period. O RhD^+^ positive (38.7%), B RhD^+^ positive (23.7%), and A RhD^+^ positive (22.3%) were most common. RhD-negative donors accounted for 8.6%, with O RhD^−^ negative comprising ~4%, critical for universal transfusion needs (Figure 1).

### 3.2 Donation trends and sex distribution

**Figure 2:**
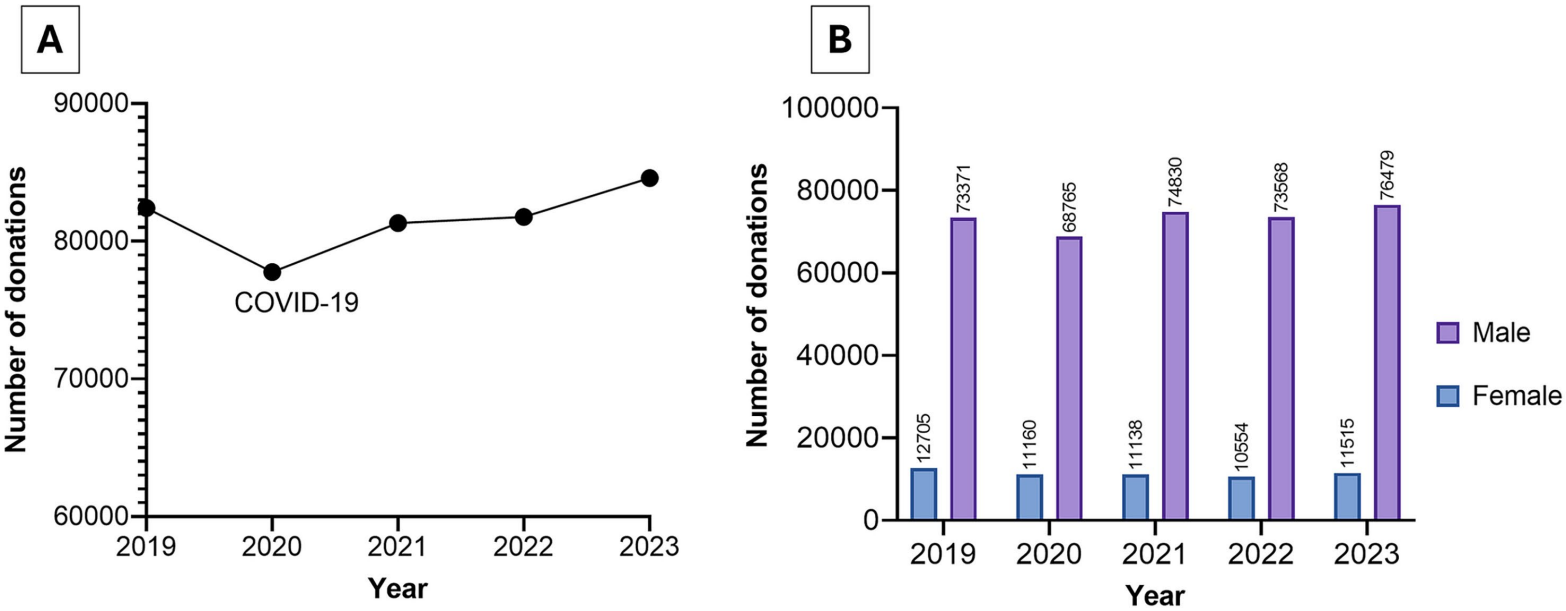
Annual donation trends and sex distribution of blood donors in Kuwait, 2019–2023. **(A)** Total number of blood donations collected annually at Kuwait Central Blood Bank. The number of donations decreased during COVID-19 and then increased steadily to 86,000 in 2023. **(B)** Distribution of donations by sex, presented as absolute counts per year. Male donors consistently accounted for >85% of all donations.

Annual donations ranged between 77,000 and 85,000. A decline was noted during the COVID-19 pandemic (2020–2021), followed by recovery to 86,000 in 2023. Male donors consistently represented >85% of the donor pool, underscoring the gender imbalance in voluntary blood donation (Figure 2).

### 3.3 Discarded blood components

**Figure 3:**
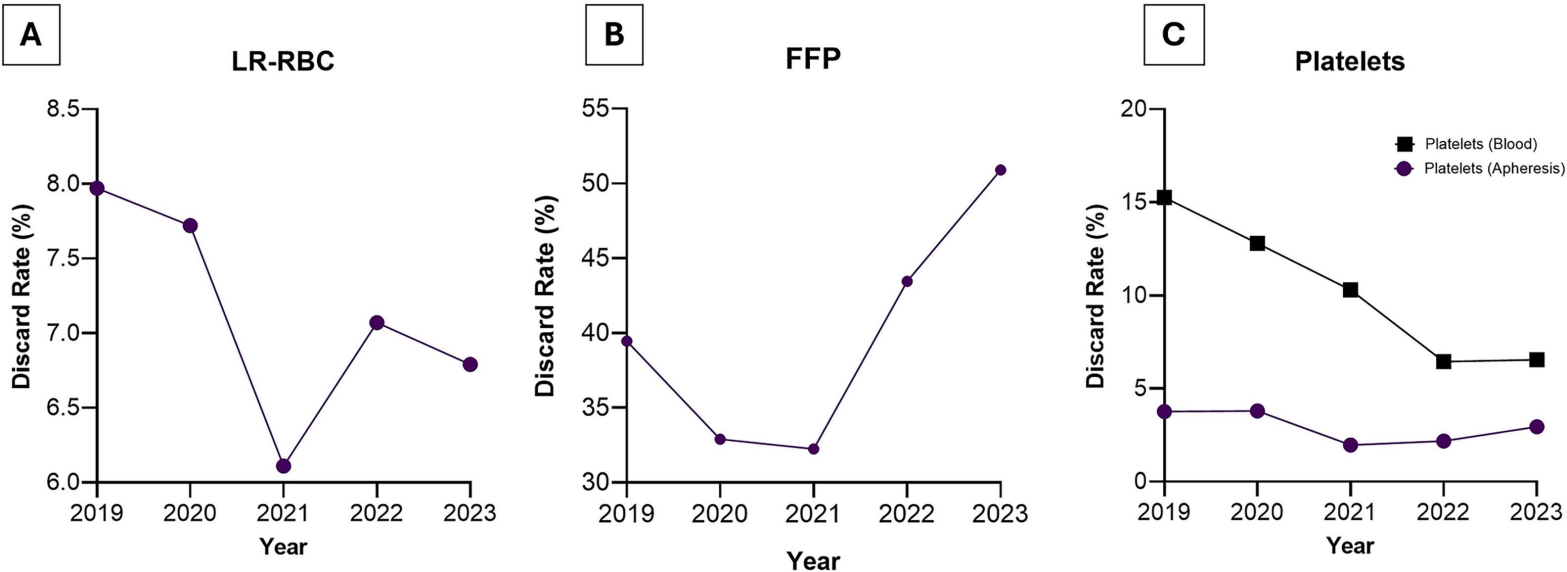
Annual number of discarded blood components at Kuwait Central Blood Bank from 2019 to 2023. Data show wastage for **(A)** LR-RBC, **(B)** FFP, and **(C)** platelets, highlighting temporal patterns and overall discard burden.

Wastage patterns varied annually, with FFP comprising the largest discard volumes (Figure 3). Lowest discard rates of FFP were observed during 2020–2021, coinciding with pandemic-related disruptions, followed by a spike in later years.

### 3.4 Transfusion-transmissible infections

**Figure 4:**
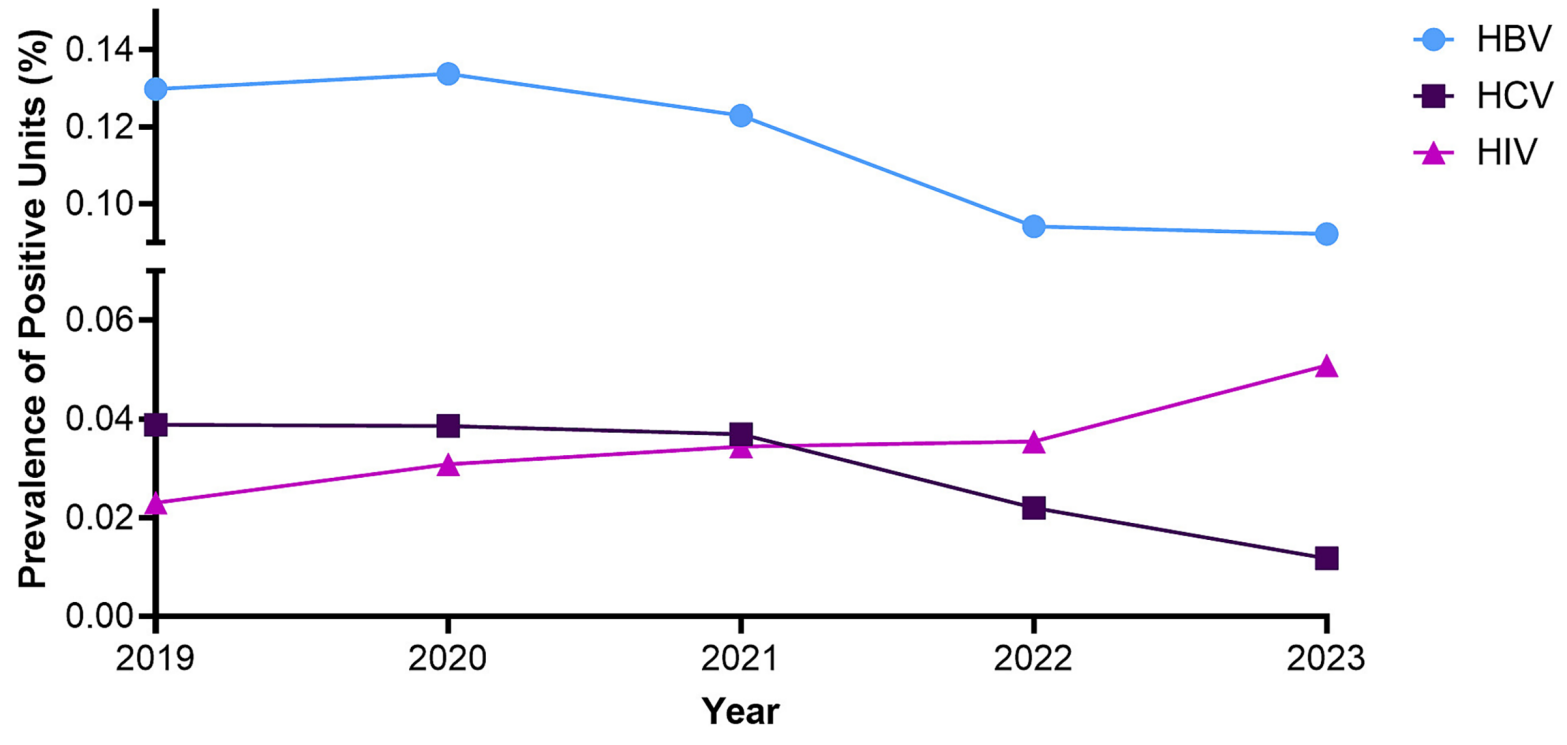
Percentage of blood donations testing reactive for hepatitis B virus (HBV), hepatitis C virus (HCV), and human immunodeficiency virus (HIV) at Kuwait Central Blood Bank during 2019–2023. Prevalence is expressed as the proportion of reactive units among total donations per year.

Nucleic acid test (NAT) screening outcomes showed variation in the prevalence of reactivity for transfusion-transmissible infections (TTIs). HBV ranged from 0.06 to 0.11% (60–110 per 100,000 donations), HCV from 0.03 to 0.08% (30–80 per 100,000 donations), and HIV from 0.02 to 0.05% (20–50 per 100,000 donations). (Figure 4).

### 3.5 Patient testing workload

**Figure 5:**
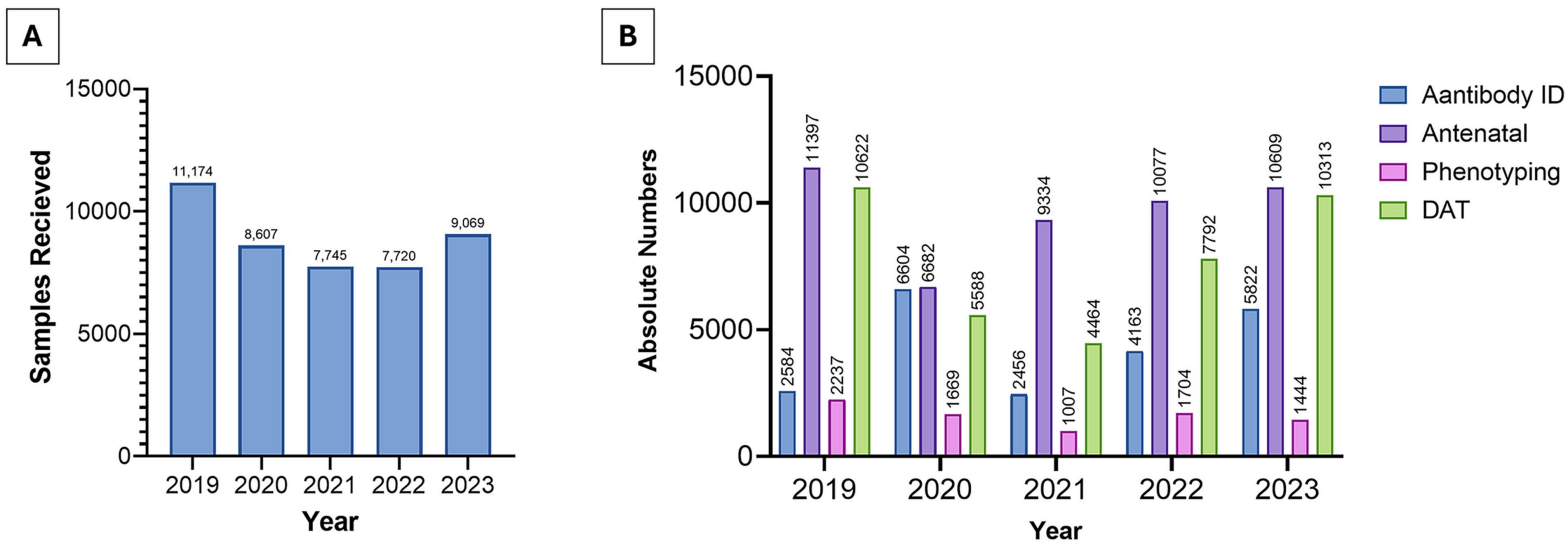
Annual patient testing workload at Kuwait Central Blood Bank, 2019–2023. **(A)** Total number of patient samples received for immunohematology investigations. **(B)** Annual distribution of antibody identification tests, antenatal testing, red cell phenotyping, and direct antiglobulin tests (DAT). Both total sample volume and testing activity declined during the COVID-19 pandemic and increased again by 2023.

The overall immunohematology workload at KCBB changed during the study period. Both the total number of patient samples received, and the number of tests performed (including antibody identification, antenatal screening, red cell phenotyping, and direct antiglobulin testing) declined during the COVID-19 pandemic years (2020–2021) with numbers showing a gradual increase in 2023 (Figure 5).

### 3.6 Prevalence of red cell antibodies in patient samples

**Figure 6:**
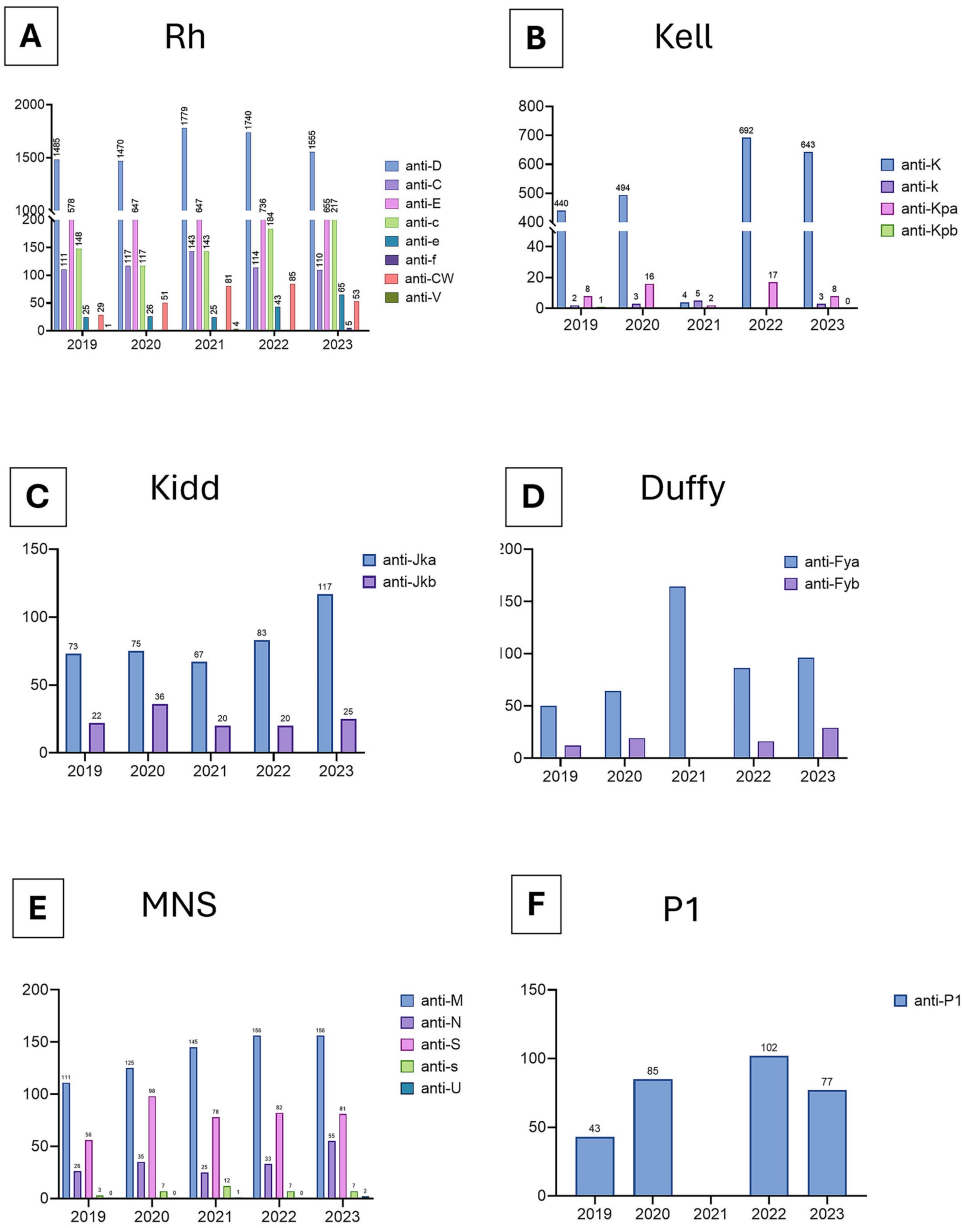
Antibodies detected in patient samples at Kuwait Central Blood Bank, 2019–2023. Data are presented as absolute counts and grouped by ISBT blood group systems: **(A)** Rh (ISBT 004), **(B)** Kell (ISBT 006), **(C)** Kidd (ISBT 009), **(D)** Duffy (ISBT008), **(E)** MNS (ISBT 002), and **(F)** P1PK (ISBT 003). Only selected blood group systems are displayed; additional antibody specificities were detected but are not shown here.

A wide spectrum of clinically significant alloantibodies was detected over the five-year period. The most frequently identified antibodies were directed against antigens in the Rh, Kell, and Kidd systems, with additional contributions from MNS and P1PK. Annual fluctuations were observed, but no marked upward or downward trend was evident (Figure 6; Table 1).

**Table 1:** Other antibody specificities identified in blood bank investigation samples (2019–2023).

Year	Rh				Lutheran		Lewis		Yt	Xg	H	Gerbich
	U	f	C^w^	V	Lu^a^	Lu^b^	Le^a^	Le^b^	Yt^a^	Xg^a^	H	Ge2
2019	–	–	29	1	5	–	74	61	–	–	–	5
2020	–	–	51	–	3	–	59	78	–	8	2	6
2021	1	–	81	4	8	–	86	80	5	–	2	3
2022	–	–	85	–	15	–	67	52	1	1	–	9
2023	2	5	53	0	7	0	62	59	0	0	2	13

–, data not reported in the annual summary (may represent 0).

### 3.7 Antibody prevalence in antenatal samples

**Figure 7:**
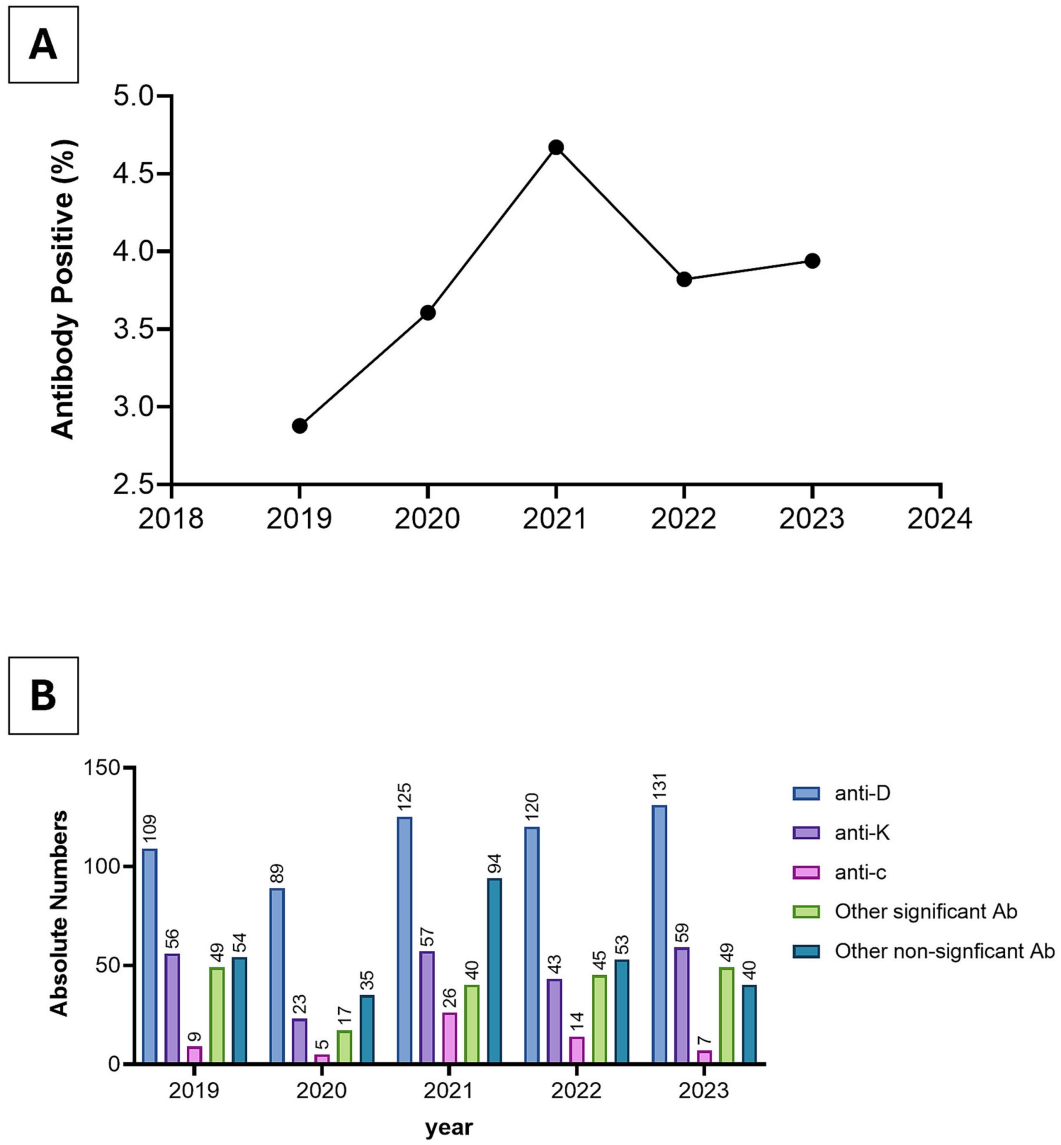
Antibody detection among antenatal samples tested at Kuwait Central Blood Bank, 2019–2023. **(A)** Percentage of antenatal samples that were antibody-positive. **(B)** Distribution of identified antibodies in positive antenatal cases, presented as absolute counts.

Among antenatal samples tested during 2019–2023, a consistent minority were antibody-positive. Most detected antibodies were directed against Rh and Kell antigens, reflecting their clinical significance in HDFN. The proportion of antibody-positive antenatal samples averaged at 3.8% over the 5 years, with a peak of 4.7% in 2021. The most prevalent antibody was anti-D, followed by anti-K (Figure 7).

## 4 Discussion

This study presents a descriptive five-year national analysis (2019–2023) of blood donation trends, donor characteristics, component utilization, identified agents of TTIs, and immunohematology workload at KCBB. By combining the donor and patient-side data, the findings provide a holistic assessment of transfusion services in Kuwait and allow benchmarking against regional and international trends.

### 4.1 Donor trends and gender distribution

Annual donations averaged at 82,000 per year, with a temporary decline during the COVID-19 pandemic followed by full recovery in 2023 (Figure 2). Male donors consistently represented more than 85% of the donor pool, reflecting patterns observed across Gulf states where cultural, social, and medical factors limit female participation. In Saudi Arabia, for instance, female donors account for only 2.5–7% of collections, depending on the region. The latest WHO global status report on blood safety and availability indicates that the median proportion of female blood donors in Africa is 22%, suggesting that low female participation is a regional issue, although still higher than the proportion observed in Kuwait (12). Although women comprise ~39% of Kuwait’s population, their contribution to blood donation remains disproportionately low, highlighting an opportunity for improvement. Targeted strategies to enhance female recruitment may therefore strengthen both the sustainability of the donor pool and the resilience of national supply (13, 14).

### 4.2 ABO and RhD distribution in Kuwait compared to other populations

The ABO and RhD distribution in Kuwait maintained similar levels across the study period, with O RhD^+^ positive (38.7%), B RhD^+^ positive (23.7%), and A RhD^+^ positive (23.1%) as the most common groups, and AB RhD^−^ negative (0.6%) as the least frequent. RhD-negative donors represented 8.6% of the donor pool (Figure 1). This pattern is consistent with other Gulf populations: in Saudi Arabia, O RhD^+^ positive was also predominant (38.7%), followed by B RhD^+^ positive (23.5%), with 11.8% RhD-negative (15); in Oman, O RhD^+^ positive was the most common (44.9%), and RhD-negative donors comprised 10.7% (16). By contrast, United States population data show 38% O RhD^+^ positive and ~15–16% RhD-negative (17), while in the United Kingdom, ~26% are RhD-negative, with O RhD^+^ positive (36%) and A RhD^+^ positive (28%) particularly emphasized in the donor pool (18). These comparisons highlight that Kuwait’s ABO profile aligns with regional Gulf norms, but the lower RhD-negative prevalence compared to Europe and North America has important implications for inventory planning and antenatal care, where RhD-negative units are critical.

### 4.3 Component utilization and wastage

LR-RBCs showed an average discard rate of 7.1% across the five-year analysis period. For platelets, discard rates averaged 2.9% for apheresis platelets and 10.3% for pooled platelets, with notable improvement over time as KCBB achieved a substantial reduction in platelet wastage. Component discard patterns fluctuated annually, with FFP consistently contributing the largest discard volumes. Discard rates were lowest in 2020–2021, coinciding with pandemic-related reductions in blood product production, but increased in subsequent years (Figure 3). Among strategies proven to reduce FFP wastage, inter-hospital redistribution of near-expiry plasma units has demonstrated substantial impact; one Canadian program, for example, prevented more than CAD 17 million in wastage through effective transfer between facilities. A 12-year study (2011–2023) from a Taiwanese teaching hospital reported that implementing stricter return-and-reissue protocols (updated in 2012) and enhanced inventory monitoring led to a marked reduction in the overall blood product wastage rate, contributing to an overall institutional average discard rate of 0.08%. However, FFP units still accounted for the highest percentage of total discarded units (36.3%), highlighting specific challenges for plasma components, such as the inability to reissue thawed plasma (20). The UK’s Blood Stock Management Scheme (BSMS) further underscores the importance of optimized stock levels, robust cold-chain procedures, and inventory management tools to minimize waste of perishable components such as FFP, particularly during supply disruptions (19–21).

As the production of blood components follows similarly structured and standardized processes, and because Kuwait has a single national center responsible for blood component manufacturing, it would be valuable to explore how applying AI based forecasting models could support more efficient production planning and reduce wastage. AI driven demand forecasting is increasingly used in manufacturing sectors with continuous production lines. Recent work has already demonstrated the potential of these tools: national level models, such as the AI based demand prediction system developed in Korea, have successfully used machine learning to forecast monthly transfusion needs, while other platforms have shown promise in reducing shortages and wastage across the blood supply chain (22, 23).

### 4.4 Transfusion-transmissible infections

When expressed per 100,000 donations, NAT screening among Kuwaiti donors showed HBV reactivity of 60–110, HCV 30–80, and HIV 20–50. In comparison, Saudi Arabia reported substantially higher HBV prevalence, 1,180 per 100,000, slightly higher HCV prevalence, 100 per 100,000, and lower HIV prevalence, 10 per 100,000. In the United Arab Emirates, donor prevalence was also higher for HBV, 234 per 100,000, and HCV, 110 per 100,000, while HIV was lower at 4 per 100,000. Data from Qatar indicated higher HBV prevalence at 300 per 100,000 and higher HCV at 600 per 100,000, although HIV prevalence was not reported. Collectively, these comparisons place Kuwait at the lower end of regional prevalence for HBV and HCV, while HIV rates are slightly higher than those observed in Saudi Arabia and the UAE (24–26). In Egypt, a recent comparative study evaluating NAT and serological screening reported HBV reactivity of approximately 710 per 100,000, HCV at 1,520 per 100,000, and HIV at 20 per 100,000; values notably higher than those observed in Gulf countries and reflecting Egypt’s well-documented burden of viral hepatitis (27). By contrast, prevalence estimates from the United States TTIMS study were markedly lower, with HBV at 5.3, HCV at 9.1, and HIV at 1.6 per 100,000 donations. Collectively, these comparisons position Kuwait’s donor reactivity rates below or comparable to most Gulf neighbors but higher than those reported from the United States, highlighting the potential for further gains in transfusion safety (11).

### 4.5 Immunohematology workload and alloantibody prevalence

The immunohematology workload at KCBB closely mirrored national healthcare activity, with total samples, antibody screens, and antenatal tests declining during the pandemic and slightly increasing again by 2023 (Figure 5). Across the study period, a wide range of clinically significant alloantibodies were identified, most frequently within the Rh, and Kell systems (Figure 6). This distribution aligns with patterns reported internationally, where antibodies against highly immunogenic antigens in these systems account for the majority of clinically relevant alloimmunization cases (28–30). These findings emphasize the ongoing need for robust antibody identification services to support transfusion safety, particularly for multi-transfused and obstetric patients.

### 4.6 Antenatal antibody prevalence

Among antenatal samples, a stable minority were antibody-positive, with Rh and Kell antibodies predominating—both clinically important for HDFN. Rates of antibody positivity in Kuwait align with estimates from high-income countries, (31, 32). Importantly, only 8.6% of the Kuwaiti donor pool is RhD-negative, a lower prevalence compared to European and North American populations (5, 18). This should be considered as the use of prophylactic anti-D policies are developed, while keeping in mind that limited availability of RhD-negative blood units amplifies the clinical and logistical challenges of managing alloimmunized RhD-negative pregnancies. These data highlight the critical importance of systematic antenatal antibody screening, effective referral pathways, and coordinated policies between obstetric and transfusion services to ensure timely access to compatible blood and optimize perinatal outcomes.

### 4.7 Strengths, limitations, and policy implications

This five-year national analysis (2019–2023) integrates donor activity (ABO/RhD distribution, TTI reactivity, and component flow) with patient immunohematology workload, providing the first consolidated benchmark from Kuwait. Limitations include reliance on aggregated reports, which prevent age or donor-status stratification, the restricted five-year window that overlaps the COVID-19 period, and incomplete or unclear reporting of some variables. Furthermore, the reliance on descriptive statistics without inferential testing means that observed trends and differences between years should be interpreted with caution and require confirmation in future studies.

Policy priorities: Raise and retain female donors by addressing iron-related deferrals and optimizing communication. Although Kuwait’s donor-deferral reasons were not captured in this dataset, evidence from nearby Gulf settings shows markedly lower female contribution and substantially higher deferral odds for women, largely from low hemoglobin but not exclusively. In a Saudi hospital cohort (10,175 presentations), women accounted for ~7% of completed donations and were ~10–12 × more likely to be deferred than men; the leading cause was low Hb, yet questionnaire factors also contributed (33). Beyond biological causes, Middle East surveys report social norms, misconceptions about eligibility, and spousal/family influence as salient barriers for women considering donation (34). Therefore, improving female participation likely requires a combined approach: medical (iron-health management for eligible donors) and culturally tailored outreach and social-marketing strategies (13, 35, 36).

Plan obstetric–transfusion pathways for RhD-negative scarcity (8.6% in our pool): Current UK/BSH guidance emphasizes systematic antenatal antibody testing, risk stratification, and coordinated access to compatible blood for HDFN, frameworks Kuwait can adapt to mitigate RhD-negative pressure (7).

Cut component wastage with structured inventory tools and redistribution. Province-wide inter-hospital transfer of near-expiry plasma/products reduced discard and costs; UK BSMS KPIs (ISI, WAPI) provide practical targets, while recent operational analyses highlight storage/repurposing failures as dominant causes, areas for SOP tightening, and forecasting (10, 37).

## 5 Conclusion

Between 2019 and 2023, the Kuwait Central Blood Bank sustained a consistent donor base and met testing demands as the principal and only national site for several blood bank investigations. Three priority areas for strengthening emerge from these data. First, the persistent male predominance in donations underscores the need for targeted strategies to recruit female donors. Second, the relatively low prevalence of RhD negative donors (8.6%) reflects both limited supply and reduced population demand. Third, variable component wastage, particularly of FFP, highlights the importance of structured inventory management, interhospital redistribution, and adoption of validated performance indicators. Addressing these gaps will improve the resilience and safety of Kuwait’s blood supply.

## Data Availability

The data analyzed in this study is subject to the following licenses/restrictions: data were extracted from officially requested annual reports of the Kuwait Central Blood Bank (KCBB) covering January 2019 to December 2023. These reports are only available in printed form upon official request through official channels in Kuwait. Requests to access these datasets should be directed to Dalal Almuaili. dalal.almuaili@ku.edu.kw.

## Footnotes

1 dalal.almuaili@ku.edu.kw.

## References

[1] World Health Organization. Global status report on blood safety and availability 2025. Geneva: World Health Organization (2025).

[2] Thakur SK Sinha AK Negi DK Singh S. Effect of COVID-19 pandemic on blood transfusion service: an experience from a regional blood transfusion center. Blood Sci. (2023) 5:209–17. doi: 10.1097/BS9.0000000000000161, 37546709 PMC10400046

[3] Chien J-h Yao C-y Chen H-f Ho T-F. Trends in blood transfusion and causes of blood wastage: a retrospective analysis in a teaching hospital. BMC Health Serv Res. (2025) 25:67. doi: 10.1186/s12913-024-12170-x, 39806373 PMC11730163

[4] Chong D Lam JCM Feng XYJ Heng ML Mok YH Chiang L-W . Blood lost: a retrospective review of blood wastage from a massive transfusion protocol in a tertiary paediatric hospital. Children. (2022) 9:1799. doi: 10.3390/children9121799, 36553244 PMC9777499

[5] Services CB. Surveillance Report 2023 (2024). Available online at: https://professionaleducation.blood.ca/en/transfusion/publications/surveillance-report (Accessed September 7, 2025).

[6] Savoia HF Parakh A Kane SC. How I manage pregnant patients who are alloimmunized to RBC antigens. Blood. (2025) 145:2275–82. doi: 10.1182/blood.2023022894, 38743880

[7] Regan F Veale K Robinson F Brennand J Massey E Qureshi H . Guideline for the investigation and management of red cell antibodies in pregnancy: a British Society for Haematology guideline. Transfus Med. (2025) 35:3–23. doi: 10.1111/tme.13098, 39912875

[8] Ahmadimanesh M Safabakhsh HR Sadeghi S. Designing an optimal model of blood logistics management with the possibility of return in the three-level blood transfusion network. BMC Health Serv Res. (2023) 23:1304. doi: 10.1186/s12913-023-10240-0, 38012729 PMC10680208

[9] Meneses M Santos D Barbosa-Póvoa A. Modelling the blood supply chain. Eur J Oper Res. (2023) 307:499–518. doi: 10.1016/j.ejor.2022.06.005

[10] Hajjaj OI Modi D Cameron T Barty R Owens W Heddle N . Reducing blood product wastage through the inter-hospital redistribution of near-outdate inventory. Transfusion. (2024) 64:1207–16. doi: 10.1111/trf.17876, 38752381

[11] Steele WR Dodd RY Notari EP Xu M Nelson D Kessler DA . Prevalence of human immunodeficiency virus, hepatitis B virus, and hepatitis C virus in United States blood donations, 2015 to 2019: the transfusion-transmissible infections monitoring system (TTIMS). Transfusion. (2020) 60:2327–39. doi: 10.1111/trf.16005, 32869326

[12] World Health Organization. Global status report on blood safety and availability 2021. Geneva: World Health Organization (2021).

[13] Almalki D Badawi MA Elgemmezi T Alabdali A Almutairi M Almogbel A . Blood donor characteristics at a hospital blood bank in Saudi Arabia: a trend-analysis. J Appl Hematol. (2024) 15:169–75. doi: 10.4103/joah.joah_74_24

[14] Information PAfC. Statistical reports – Population, gender by nationality and age, and gender distribution, Kuwait: Public Authority for Civil Information (2023). Available online at: https://stat.paci.gov.kw/englishreports/ (Accessed September 1, 2025).

[15] Eltayeb R. Frequency of ABO and rh blood groups among blood donors in the hail region of Saudi Arabia. Cureus. (2024) 16:e69195. doi: 10.7759/cureus.69195, 39398823 PMC11469338

[16] Al-Riyami AZ Al-Marhoobi A Al-Hosni S Al Mahrooqi S Schmidt M O'Brien S . Prevalence of red blood cell major blood group antigens and phenotypes among Omani blood donors. Oman Med J. (2019) 34:496–503. doi: 10.5001/omj.2019.92, 31745413 PMC6851071

[17] Cross AR. Blood types and percentages in the U.S. population. United States: American Red Cross (2024).

[18] Blood and Transplant. Blood Types – Why Give Blood. United Kingdom: NHS Blood and Transplant (2025).

[19] Heddle NM Acker JP Chassé M. Reducing blood product wastage through the inter-hospital redistribution of near-outdate inventory. Transfusion. (2024) 64:987–95. doi: 10.1111/trf.17876 38752381

[20] Lin H-C Hsu Y-T Chou Y-C. Trends in blood transfusion and causes of blood wastage: A retrospective analysis in a teaching hospital. BMC Health Serv Res. 25:12170. doi: 10.1186/s12913-024-12170-x PMC11730163 39806373

[21] Blood and Transplant. BSMS inventory management best practice review – September 2024. United Kingdom: NHS Blood and Transplant (2024).

[22] Ben Elmir W Hemmak A Senouci B. Smart platform for data blood bank management: forecasting demand in blood supply chain using machine learning. Information. (2023) 14:31. doi: 10.3390/info14010031

[23] Kwon HJ Park S Park YH Baik SM Park DJ. Development of blood demand prediction model using artificial intelligence based on national public big data. Digit Health. (2024) 10:20552076231224245. doi: 10.1177/20552076231224245, 38250146 PMC10798124

[24] Alsughayyir J Almalki Y Alburayk I Alalshaik M Aljoni I Kandel M . Prevalence of transfusion-transmitted infections in Saudi Arabia blood donors: a nationwide, cross-sectional study. Saudi Med J. (2022) 43:1363–72. doi: 10.15537/smj.2022.43.12.20220634, 36517064 PMC9994514

[25] Al Shaer L AbdulRahman M John TJ AlHashimi A. Trends in prevalence, incidence, and residual risk of major transfusion-transmissible viral infections in United Arab Emirates blood donors: impact of individual-donation nucleic acid testing, 2004 through 2009. Transfusion. (2012) 52:2300–9. doi: 10.1111/j.1537-2995.2012.03740.x, 22691239

[26] Aabdien M Selim N Himatt S Hmissi S Merenkov Z AlKubaisi N . Prevalence and trends of transfusion transmissible infections among blood donors in the State of Qatar, 2013–2017. BMC Infect Dis. (2020) 20:617. doi: 10.1186/s12879-020-05344-5, 32819294 PMC7441652

[27] Shahin D Aly R Ghannam M Khaled O Sadeq M Elzeiny A . A comparative analysis between NAT and chemiluminescence in detection of transfusion transmitted viruses in two main university blood transfusion centers. Sci Rep. (2025) 15:20109. doi: 10.1038/s41598-025-03506-6, 40541994 PMC12181300

[28] Liu C Grossman BJ. Antibody of undetermined specificity: frequency, laboratory features, and natural history. Transfusion. (2013) 53:931–8. doi: 10.1111/trf.12070, 23305295

[29] Devi KM Nepram L Hajong R Bhattacharyya D. Prevalence and specificity of red blood cell alloantibodies in patients in a tertiary Care Center in Meghalaya, India. Cureus. (2025) 17:e86347. doi: 10.7759/cureus.86347, 40688858 PMC12275498

[30] Mushkbar M Watkins E Doughty H. A UK single-Centre survey of red cell antibodies in adult patients undergoing liver transplantation. Vox Sang. (2013) 105:341–5. doi: 10.1111/vox.12059, 23763654

[31] Sugrue RP Moise KJ Federspiel JJ Abels E Louie JZ Chen Z . Maternal red blood cell alloimmunization prevalence in the United States. Blood Adv. (2024) 8:4311–9. doi: 10.1182/bloodadvances.2023012241, 38662646 PMC11372799

[32] Koelewijn J Vrijkotte T van der Schoot C Bonsel G de Haas M. Risk factors for the presence of non-rhesus D red blood cell antibodies in pregnancy. BJOG Int J Obstet Gynaecol. (2008) 115:589–96. doi: 10.1111/j.1471-0528.2008.01984.x 19210505

[33] Badawi MA Mansory EM Al-Malki A Abbas SA Mutmi H Gholam K . Exploring women's capability to donate blood in a Saudi blood bank: a COM-B model study. Medicine (Baltimore). (2025) 104:e43479. doi: 10.1097/MD.0000000000043479, 40696612 PMC12282721

[34] Goldsmith AJ Gebril NM Thull DS Abdella YE Barski L Malgor PY . A knowledge, attitudes, and practices survey concerning blood donation among Libyans. Global Journal of Transfusion Medicine. (2023) 8:17–22. doi: 10.4103/gjtm.gjtm_78_22

[35] Meulenbeld A Ramondt S Sweegers MG Quee FA Prinsze FJ Hoogendijk EO . Effectiveness of ferritin-guided donation intervals in whole-blood donors in the Netherlands (FIND'EM): a stepped-wedge cluster-randomised trial. Lancet. (2024) 404:31–43. doi: 10.1016/S0140-6736(24)01085-7, 38880108

[36] Al Shaer L Sharma R AbdulRahman M. Analysis of blood donor pre-donation deferral in Dubai: characteristics and reasons. J Blood Med. (2017) 8:55–60. doi: 10.2147/JBM.S135191, 28579846 PMC5449161

[37] Blood Stocks Management Scheme. Reporting and monitoring - Blood Stocks Management Scheme 2025. Available online at: https://www.bloodstocks.co.uk/reporting-and-monitoring/ (Accessed September 7, 2025).

